# Young women’s perspectives on unwanted sexual attention: navigating boundaries, responsibility, and prevention – a qualitative study

**DOI:** 10.1080/17482631.2026.2640189

**Published:** 2026-03-06

**Authors:** Elin Salemonsen, Agnes C. Wilhelmsen Giertsen, Malene Seth

**Affiliations:** a Department of Health and Caring Science, Western Norway University of Applied Sciences, Faculty of Health and Social Sciences, Masters in Midwifery and Public Health Nursing, Bergen, Norway; b Ålesund Municipality, School Health Services, Ålesund, Norway

**Keywords:** Boundaries, sexual health, sexual harassment, young women, unwanted sexual attention

## Abstract

**Purpose:**

Norwegian youth report experiences of unwanted sexual attention (UWSA), which can negatively affect sexual health, well-being and quality of life. The aim of this study was to explore young women’s perceptions of UWSA in Norway and their views on how it can be prevented and managed.

**Method:**

This study employed an exploratory and descriptive design, based on nine individual interviews with young women aged 16–23.

**Results:**

Three overarching categories were identified: 1) Individual perceptions of severity and consequences of UWSA—towards normalization, 2) The importance of looking out for one another, the courage to speak up, and possessing the capacity for action in situational contexts; and 3) The need for increased knowledge on sexual health, sexual rights, and boundary-setting.

**Conclusion:**

The study underscores the need to strengthen sexuality education, emphasizing sexual rights, respect for personal boundaries, and young people's ability to act appropriately to UWSA. Challenging gender stereotypes and the normalization of UWSA is crucial. Expanded collaboration between schools and school health services on structured, student-active programs, including training in how to respond to UWSA, may help to prevent sexual harassment. Preventive efforts must be politically and organizationally anchored, with responsibility understood as collective rather than solely individual.

## 1. Introduction

Norwegian adolescents, particularly young women, report experiencing unwanted sexual attention (UWSA) or sexual harassment, which includes sexual comments, unwanted touching, the sharing of sexualised images, rumour- spreading, or pressure to engage in intercourse or other sexual acts (Bakken, 2024; Kripos, 2022). Many adolescents are active on social media, where such violations frequently occur (Ødegård, 2022; Pedersen et al., 2023). Over 60% of young women aged 16–17 in Norway consider UWSA to be an increasing problem, while also noting that it has become easier to report such incidents (Ødegård, 2022; Skoog et al., 2023).

The Norwegian Ungdata (Young data) survey revealed that 18% of girls reported experiencing unwanted sexual touching, compared to 8% of boys. In upper secondary school, 11% of girls reported being pressured into sexual acts, while 3% of boys reported the same. Additionally, 6% of girls and 4% of boys reported that sexualised images or videos of them had been shared without their consent. Verbal UWSA is common as early as 8^th^ grade (age 13-14), and unwanted touching increases with age (Bakken, 2024). Studies from the Norwegian *Ung i Oslo (Young in Oslo)* have shown that girls in lower and upper secondary school are particularly vulnerable to sexual harassment (Bakken, 2023). A Norwegian study identified a link between exposure and maturation (Frøyland, 2023), and an American study found that sexual harassment often occurs in school settings, especially in hallways, but also in classrooms, locker rooms, gyms, and outside school areas (Espelage et al., 2016).

UWSA represent a serious societal issue with significant consequences for health, well-being and quality of life, including reduced body image and self-esteem, anxiety, and depression (Bendixen et al., 2018; Nisja et al., 2021; Skoog & Kapetanovic, 2023). Research has demonstrated a connection between sexual harassment and emotional problems, negatively affecting both psychological and sexual development Skoog and Kapetanovic (2023), and contributing to poorer psychosocial health among affected youth (Bonsaksen et al., 2024). Adolescents who experience digital sexual violations often report feelings of shame and guilt (Kripos, 2022). They also report increased exposure to violence, physical sexual harassment, health problems, and risk behaviours, such as smoking and excessive alcohol consumption. Exposure levels are higher among adolescents who are frequent users of social media, particularly those who feel pressure related to the number of “likes” and followers on digital platforms (Frøyland, 2023).

Unwanted sexual attention (UWSA) is defined in the Norwegian Equality and Anti-Discrimination Act §13 as sexual harassment if “it has the purpose or effect of being offensive, frightening, hostile, degrading, humiliating or bothersome” (Norwegian Ministry of Culture and Equality, 2018). Sexual harassment may include sexual comments about body and appearance, simulation of sexual movements, groping, touching, and assault, as well as the display of videos or images with sexualised content (The Equality and Anti-Discrimination Ombud (LDO), n.d.). This study consistently uses the term UWSA as it is considered a broader concept than sexual harassment.

Sexual health is defined as physical, emotional, mental, and social well-being related to sexuality. It is a resource and protective factor that promotes quality of life and coping (Norwegian Ministry of Health and Care Services, 2016; Norwegian Diractorate of Health, 2023), and it presupposes the safeguarding of sexual rights. Central to sexual health are the rights to bodily autonomy, freedom from violence, access to sexuality education, and the right to choose whether to be sexually active (Norwegian Ministry of Health and Care Services, 2016). Knowledge of rights, respect, and boundaries is essential for developing personal autonomy while also respecting the boundaries of others (Norwegian Ministry of Health and Care Services, 2016). The Norwegian national strategy for *Sexual Health 2017–2022*—*Let’s Talk About It!* recommends integrating sexual health and rights into local public health initiatives (Norwegian Diractorate of Health, 2020). Municipalities are responsible for ensuring that all children and young people have access to comprehensive sexuality education in kindergarten and school, aligned with national frameworks and curricula (Ministry of Health and Care Services, 2016; Norwegian Diractorate of Health, 2020). Topics related to the body, boundaries, and sexuality are included across several school subjects and form part of the interdisciplinary theme of public health and life skills (Norwegian Directorate for EducationTraining, 2020).

Public child health clinics and school health services in Norwegian primary care are strongly recommended to promote sexual health. Child health clinics are advised to provide guidance so that parents have sufficient knowledge to talk to their children about the body, gender, sexuality, safety, and boundaries. School health services should contribute to the school’s relationship- and sexuality education, particularly in the area of sexual health (Norwegian Diractorate of Health, 2017). For sexuality-education to have a health-promoting and preventive effect, it is essential that children and adolescents receive the necessary knowledge before they encounter situations in which it is needed (Norwegian Ministry of Health and Care Services, 2016).

Many adolescents in Norway perceive school-based sexuality education as inadequate (Astle et al., 2021; Helbekkmo et al., 2021; Sex and Society, 2022). A review study highlights the need to reduce stigma, increase relevance, and ensure safe learning environments with open and non-judgmental teachers to improve sexuality education (Corcoran et al., 2020). National research shows that adolescents in Norway call for more practical and concrete knowledge about sexuality (Helbekkmo et al., 2021).

Although substantial international research has examined young women’s experiences of sexual harassment, there is limited national and international knowledge about their perceptions of UWSA and their views on how such situations can be prevented and managed. In Norway, various educational programmes have been developed to prevent sexual harassment, yet young women continue to express a need for more knowledge on the topic, as shown in a Norwegian study by Goldschmidt-Gjerløw (2022). The increasing number of reported UWSA in Norway (Bakken, 2023, 2024) underscores the need for broader and structural measures. Despite this increase, few qualitative studies have explored young women’s perceptions of the phenomenon. Gaining insight into their perspectives is therefore crucial to inform the development of relevant educational programmes that can prevent sexual harassment and promote positive sexuality, sexual health, and well-being. The aim of the study was to explore how young women in Norway perceive UWSA and how they believe such attention can be prevented and managed. The following research question is posed:

What are the perceptions of young women in Norway regarding unwanted sexual attention (UWSA), and how do they believe it can be prevented and managed?

## 2. Materials and methods

### 2.1. Design

This study employed an exploratory and descriptive design with an inductive approach (Polit & Beck, 2021). Individual interviews were conducted with young women to explore their personal perceptions of the phenomenon of unwanted sexual attention (UWSA).

### 2.2. Participants and recruitment

A purposive sampling method was used to ensure that participants had relevant experience with the phenomenon under investigation (Polit & Beck, 2021). Inclusion criteria consisted of women aged 16–25, who were capable of providing informed consent. A total of nine young women aged 16–23 participated, with a mean age of 19.5 years (Table I). The participants were recruited through managers affiliated with the school health services at upper secondary schools and youth health clinics in a municipality in Western Norway. The last author (MS) shared information about the study at schools and through flyers placed in the waiting areas of youth health clinics.

**Table I.** Participant characteristics.

Participant	Age	Recruit setting	Cultural background	Habitat	Occupational status
1	20	Youth Health Clinic	Norwegian	Urban	Student
2	17	Upper Secondary school	Middle East	Rural	Pupil
3	16	Upper Secondary school	Norwegian	Rural	Pupil
4	22	Youth Health Clinic	Norwegian	Urban	Student
5	22	Youth Health Clinic	Norwegian	Urban	Student
6	18	Upper Secondary school	Norwegian	Urban	Pupil
7	22	Youth Health Clinic	Norwegian	Urban	Student
8	23	Youth Health Clinic	Norwegian	Urban	Student
9	16	Upper Secondary school	Norwegian	Urban	Pupil

### 2.3. Data collection

A semi-structured interview guide was developed by the authors (ES, AG, MS) in accordance with Kvale and Brinkmann (Kvale & Brinkmann, 2015) to explore how the young women perceive UWSA and their views on prevention and management (Table II). The interviews were conducted in an open format, allowing for elaboration and follow-up on the participants` statements. A total of nine individual interviews were conducted by MS, primarily at various educational institutions, with one interview conducted digitally via Microsoft Teams. The study purpose was outlined, and the last author provided a brief account of her professional background as a PHN along with her clinical interests. The duration of the interviews ranged from 30 to 75 minutes. All interviews were audio-recorded and transcribed verbatim in Norwegian by MS. The recordings were subsequently deleted, and all transcripts were anonymized to remove any identifying information.

**Table II.** Thematic guide for individual interviews.

How do you understand digital, verbal, and physical sexual advances? What are your thoughts on individuals who approach others in unwanted ways? What do you think the consequences of unwanted verbal, digital or physical sexual advances might be? To what extent do you perceive unwanted verbal sexual advances as common? How do you think such advances can be handled or responded to? If you were subjected to such advances, how do you think you would react? If you believe the education could be improved, what changes would you suggest? Are there aspects of society or the upbringing environment that could promote positive sexual health among youth?

The data were reviewed by all authors to assess their relevance to the research question. The concept of information power by Malterud et al. (2016) was used to evaluate the adequacy of the sample size. Given the narrow scope of the research question, the sensitivity of the topic, and the richness and quality of the data (Malterud et al., 2016), the sample was deemed sufficient.

### 2.4. Data analysis

The interviews were analysed using qualitative content analysis (Graneheim & Lundman, 2004). Initially, the data were read thoroughly to gain an overall understanding. A matrix was developed in Microsoft Word to systematically organise the material. Meaningful units were identified in each interview, then condensed and coded. The initial analysis and coding were conducted by ES and MS. Through collaborative discussions among all the authors, the codes were grouped into subcategories and subsequently into overarching categories. The final manifest categories (Graneheim & Lundman, 2004; Graneheim et al., 2017; Lindgren et al., 2020) present the study’s primary findings and offer a descriptive response to the research question regarding how young women perceive UWSA and how they believe it can be prevented and managed.

### 2.5. Reflexivity and rigour

Throughout the research process, the authors maintained a reflexive stance, acknowledging how their own backgrounds, values, and professional roles may have influenced the study. All three authors are female public health nurses with extensive experience working in school health services and youth health clinics. This professional background provided valuable contextual insight into the phenomenon under investigation, while also necessitating a reflexive approach throughout the research process. To enhance reflexivity, the development of the interview guide and the interpretation of the data were conducted collaboratively, with ongoing discussions to challenge assumptions and ensure that the participants’ voices were accurately represented. All authors engaged in critical dialogue during coding and categorisation to promote transparency, and efforts were made to remain open to alternative interpretations. Reflexivity was further supported through memo writing and iterative reviews of the data in relation to the research question.

### 2.6. Ethical considerations

The study adhered the ethical principle outlined in the Declaration of Helsinki (World Medical Association (WMA), 2024). The participants received written information about the study, including details on how the interview data would be handled, and their right to withdraw at any point. All participants provided written informed consent. In accordance with Norwegian legislation, individuals aged 16 and above are legally permitted to provide independent consent to participate in health research, provided the study does not involve clinical interventions or drug trials (Norwegian Ministry of Health and Care Services, 2024). Therefore, parental consent was not required. Based on national legislation, the study was approved by Norwegian Centre for Research Data (Ref. no. 540314) and the Regional Committees for Medical and Health Research Ethics (Ref. no. 490016). The participants were asked about their perceptions of unwanted sexual attention, rather than their personal experiences. They were also informed that they could contact their general practitioner or the school nurse or Youth Health Clinics if they experienced any emotional reactions following the interviews and needed support.

## 3. Results

The analysis resulted in three overarching categories: 1) Individual perceptions of the severity and consequences of UWSA—towards normalisation, 2) The importance of looking out for one another, having the courage to speak up, and possessing the capacity to act in situational contexts; and 3) The need for increased knowledge on sexual health, sexual rights, and boundary-setting. The results are presented according to these categories and their respective subcategories (Table III).

**Table III.** Categories and subcategories.

Category	Individual perceptions of the severity and consequences of UWSA –towards normalisation	The importance of looking out for one another, having the courage to speak up, and possessing the capacity to act in situational contexts	The need for increased knowledge on sexual health, sexual rights, and boundary-setting
**Subcategory**	Differences between digital, verbal, and physical unwanted sexual attention	Reactions and personal capacity to act	Knowledge about the consequences of UWSA must be communicated
	Unwanted sexual attention as a threat to integrity and mental health	Shared responsibility to look out for one another	Greater emphasis on respect, attitudes, boundary-setting, and sexual rights in education—from early childhood to upper secondary school
	Sexual attention can be perceived positively	The courage to speak out	The need for knowledge on the practical handling of UWSA

### 3.1. Individual perceptions of the severity and consequences of UWSA - towards normalisation

The young women in the study expressed diverse views on unwanted sexual attention (UWSA) and its various forms, with individual boundaries regarding what constitutes acceptable sexual attention. While some perceived UWSA as harmless and trivial, the majority described it as unacceptable, harassing, and a threat to personal integrity, with potentially serious consequences for mental and sexual health. Most of the participants believed that men are often the perpetrators of unwanted advances, while also acknowledging that men can be victims too, though such experiences are less frequently reported. Several participants expressed concern that this behaviour may become normalised if not met with consequences. At the same time, some young women noted that certain forms of sexual attention could be perceived as flattering or as a compliment.

#### 3.1.1. Differences between digital, verbal, and physical unwanted sexual advances

UWSA was described as prevalent in contemporary society, particularly in its digital and verbal forms. Some participants felt that a culture has emerged in which sexual attention is increasingly accepted. None of the forms of UWSA were considered acceptable, however, physical unwanted attention, such as inappropriate touching, was viewed as more serious than verbal or digital advances. The young women suggested that such behaviour may stem from a need for attention, insecurity, upbringing, attitudes, or problematic social norms:

*“I wonder what’s going on in their heads and what they’ve learned growing up. They should get help. They want attention and go about it the wrong way.”* (Participant 6, age 18)

Digital UWSA was described as unsolicited messages or images with sexual content, such as “dick pics” or “nudes”, sent via social media, online platforms, or mobile phones, often anonymously and difficult to protect oneself against. The participants believed that people tend to be bolder behind a screen and that digital UWSA is more common than face-to-face verbal advances. Several expressed concern that this trend is becoming normalised:

*“It’s stupid that people do this, but also very easy and very common. It’s kind of something you’re just supposed to accept because it’s so normal. On Snapchat, I feel like there’s a lot of it, and on those dating apps, it quickly turns into sexual comments… and you’re just supposed to like it…”* (Participant 7, age 22)

Verbal UWSA was described as direct, face-to-face remarks with sexual content, such as catcalling or sexually charged utterances. The young women felt that verbal UWSA could be experienced as harassment, and that alcohol lowers the threshold for such behaviour. Some believed that men may use such language to appear cool or because they think it’s how compliments are given.

Physical UWSA was primarily perceived as touching or being touched in a sexually suggestive manner. Some described it as being groped or fondled. All the young women considered physical UWSA the most serious form, more frightening, disrespectful, and boundary-crossing than verbal or digital UWSA:

*“… it can feel very violating… I believe it’s experienced as much more dramatic and serious when someone crosses physical boundaries than verbal or digital ones.”* (Participant 7, age 22)

Some participants noted that groping is particularly common at parties involving alcohol, while another indicated that it has become so normalised that one simply has to accept it. A few speculated that such behaviour might be an awkward attempt to connect with someone they find attractive. Many emphasised that UWSA should have consequences, though the severity depends on the form, location on the body, extent, context, and in some cases, the identity of the perpetrator.

#### 3.1.2. Unwanted sexual attention as a threat to integrity and mental health

The young women believed that the severity of UWSA could negatively affect self-image, as well as mental- and sexual health and well-being. They described all forms of UWSA as potentially disrespectful and threatening, leading to discomfort, fear, and a sense of being objectified. Having one’s boundaries violated was seen as an attack on personal integrity:

*“… it quickly becomes disrespectful—I become a thing and not a person.”* (Participant 4, age 22)

Several participants felt that unsolicited sexual images or messages could be more distressing than words, and that normalisation of such behaviour may blur boundaries, especially among children and adolescents. They also noted that such images, whether of oneself or others, could have serious consequences, including body image pressure, insecurity, anxiety, social withdrawal, and poor sexual health, potentially even ruining a person’s life:

*“If it’s pictures being sent, I think it causes more harm than words. I think it’s hard to forget and can be damaging in that sense. It can make someone not want to go to school, avoid social gatherings, and develop anxiety when interacting with people…”* (Participant 6, age 18)

The consequences of *verbal UWSA* were described primarily as causing confusion, fear, and anger. Sexual comments about one’s body, appearance, or clothing, when unsolicited, were seen as uncomfortable and objectifying, affecting self-image, body image, and behaviour, such as clothing choices or feelings of safety in public spaces. The severity was assessed based on context and whether the person was alone:

*“It really depends on the setting, I think, and how you receive it… It’s not pleasant to get a sexual comment if you didn’t ask for it… It can make you feel more insecure about going where you used to go, avoiding the place, and you might start wondering what’s next.”* (Participant 8, age 23)

Physical UWSA was considered the most frightening, with potentially serious consequences for mental health. Some participants believed it could lead to anxiety, eating disorders, a need for control, social withdrawal, and negative views on sexuality. One participant described how it could erode trust in men and make conversations about sex uncomfortable:

*“They might feel uncomfortable being around men… that they develop negative thoughts about sexual health, or sexual matters in general. That they just feel uncomfortable when people talk about it or when it’s mentioned. Maybe the person becomes afraid to be alone with someone too.”* (Participant 2, age 17)

#### 3.1.3. Sexual attention can be perceived as positive

The young women had varying boundaries for what they considered unwanted sexual attention, and assessed situations differently depending on context, relationship, and intent. Some participants expressed that verbal or digital sexual advances were not necessarily perceived as unpleasant if they could be interpreted as positive comments about appearance or body. One participant said:

*“(…) it can also be meant as a compliment from the other person.”* (Participant 7, age 22)

Some of the young women felt that sexual advances could be flattering and boost self-confidence. A few believed that some persons might enjoy receiving sexualised images, even if they had not asked for them.

### 3.2. The importance of looking out for one another, having the courage to speak up, and possessing the capacity to act in situational contexts

The young women emphasised the importance of being accompanied by others, particularly in contexts where the risk of verbal or physical UWSA is heightened, such as in settings involving alcohol. They considered it essential to have the courage to speak up, both on behalf of oneself and others who may be subjected to UWSA. Taking necessary precautions and possessing the ability to act in such situations were regarded as critical. Several participants noted that many adopt a passive stance, which often places the burden of responsibility on the individual.

#### 3.2.1. Reactions and personal capacity to act

The young women expressed varying views on how they might respond to *digital sexual advances*. While some assumed they would not be significantly impacted by receiving sexualised images, others said they would feel unsettled, though not necessarily deeply affected:

*“I think I would find it unpleasant, but maybe not think too much about it. I don’t think I would take it too personally.”* (Participant 5, age 22)

Regardless of their initial reactions, most participants stated they would reject the sender, delete or block the person, and some would report the incident. If images of themselves were shared without consent, they would contact the police.

In relation to *verbal or physical UWSA*, many were uncertain about how they would react in the moment. Most believed they would feel uncomfortable and uncertain, possibly lacking the courage to speak up. They believed their responses would depend on the context, the relationship with the perpetrator, and whether they were alone. Several noted that alcohol might lower the threshold for speaking up, and that it would be easier to do so if accompanied by others. In situations involving physical advances, especially at night and unaccompanied, reactions were expected to range from fear and paralysis to anger and physical response. Some feared that speaking out could escalate the situation:

*“I don’t think I would dare to speak up. I’d probably smile and kind of play along, because you get caught off guard and don’t know what the other person might do if you oppose them…”* (Participant 7, age 22)

Most participants felt it was important to try to speak up or leave the situation if possible, and many said they would seek safer spaces or contact the police. They also felt it was necessary to take precautions, such as avoiding certain places alone at night or choosing clothing carefully, even though such measures were perceived as unfair and restrictive. One participant said:

*“I wear a long coat if I’m going out and it’s getting late. It’s good to have men with you or to walk in large groups.”* (Participant 6, age 18)

#### 3.2.2. Shared responsibility to look out for one another

Most of the young women also emphasised the importance of looking out for one another in situations where verbal or physical UWSA may occur. They believed individuals are more vulnerable when alone and that mutual support is essential, including seeking help from others or contacting the police if necessary. Party settings with high levels of alcohol consumption were seen as particularly risky, and participants suggested that friends should ally and agree in advance, deciding that on person would consume less alcohol and assume responsibility for monitoring and assisting if needed. As one participant put it:

*“(…) if someone get punched, you would naturally try to help them (…) it’s the same. You have to try to help.”* (Participant 4, age 22)

#### 3.2.3. Courage to speak up

The young women emphasised the importance to speak up in situations involving unwanted sexual advances, asserting that the responsibility to intervene lies both with the person affected and with those who witness such incidents. Several highlighted the need to support one another and to clearly signal that such behaviour is unacceptable, even when it involves friends. Speaking up was seen as crucial for setting boundaries and preventing UWSA. Intervention by bystanders was considered important in both verbal and physical instances:

*“You can stop it yourself, but I don’t think it’s always that easy. I believe those around you have the most power to stop it. It’s probably easier for outsiders to see that something inappropriate is happening to someone, so I think it can be handled by others stepping in as well.”* (Participant 7, age 22)

The participants viewed it as a shared responsibility to help in such situations, but some felt that many avoid conflict and therefore refrain from intervening or offering support:

*“It’s like no one does anything about it, you kind of have to take responsibility yourself and leave the situation or something. No one does anything. Most people remain silent and accept that it happens.”* (Participant 4, age 22)

### 3.3. The need for increased knowledge on sexual health, sexual rights, and boundary-setting

The young women expressed a clear need for more comprehensive knowledge about sexual health, including the consequences of UWSA, as well as education on boundary-setting and sexual rights. They emphasised the importance of addressing the issue of UWSA public discourse, promoting respectful attitudes and values, and increasing awareness of how such situations can be handled in practice.

#### 3.3.1. Knowledge about the consequences of UWSA must be communicated

The young women underscored the importance of addressing the issue of UWSA and raising awareness about the consequences through various platforms, including social media such as TikTok and Snapchat, as well as news outlets. Several participants called for more information and education in schools about the emotional and legal consequences of UWSA. Regarding the sharing of images, they stressed the need for improved digital literacy and clearer messaging that such actions are serious, even if they are common:

*“... I think there should be more focus on the consequences of crossing someone’s boundaries. That people reflect a bit—like, how would you feel if someone did that to you? That it’s mirrored a bit.”* (Participant 7, age 22)

#### 3.3.2. Greater emphasis on respect, attitudes, boundary-setting, and sexual rights in education—from early childhood to upper secondary school

The young women emphasised that sexual rights should apply equally to all individuals, regardless of gender, and that this must be taught early, both within families and in educational institutions. They believed that the education they had received was primarily biological in nature, with topics such as consent, respect, attitudes, and boundary-setting either superficially addressed or omitted entirely. They advocated for a continuous focus on sexual health, rights, and respect for personal boundaries throughout the educational pathway, from early childhood education to upper secondary school, as a means of preventing UWSA:

*“I think it’s good that they start with boundaries and such early (...) I definitely don’t think it’s enough to start in lower secondary school (...) I think it needs to continue all the way through.”* (Participant 9, age 16)

Most participants believed that both schools and families must contribute to teaching young people that it is acceptable to set boundaries, for themselves and toward others. Several emphasised that education on boundary-setting should be practical and nuanced, equipping youth with tools to interpret others’ signals and express their own limits:

*“You have to dare to speak up, because the other person can’t read your mind. So you kind of have to be able to say what you want and what you don’t want. But to do that, you need to have learned what boundaries are and where they should be, and then use that knowledge to say no and yes.”* (Participant 4, age 22)

Several of the young women felt that school health nurses are better suited than teachers to promote positive sexuality, and that more engaging digital resources should be developed to reach young people effectively.

#### 3.3.3. The need for knowledge on the practical handling of UWSA

The young women expressed a need for more concrete knowledge on how to respond to UWSA and how to practically handle situations particularly involving verbal and physical advances. Most participants believed that it should be easier to report or file complaints against individuals sending sexualised images, and that consequences for such behaviour should be more severe. Several participants suggested that moderation in alcohol consumption should be encouraged among youth. They also emphasised the importance of bystanders taking responsibility and intervening when someone is subjected to UWSA.

Responsibility for speaking up and offering help was seen as both individual and collective, though many noted that people often hesitate to act. This reluctance was attributed by several participants to a lack of education on how to respond in such situations. They proposed that schools should include training on how to support others and intervene when someone is experiencing UWSA. Additionally, several participants suggested that professionals should more effectively utilise social media to disseminate knowledge about sexual rights and how to handle sexual harassment in a credible and engaging way:

*“I think TikTok is a good channel… it's short and to the point. Instagram and Snapchat can work for certain things. It’s hard to get people to read it without fun headlines because it’s so difficult to catch people’s attention!”* (Participant 4, age 22)

## 4. Discussion

The aim of this study was to explore young women’s perceptions of UWSA and their views on how it can be prevented and managed. This discussion highlights the main findings: the normalisation of UWSA and how it occurs, the importance of bystander education and both individual and collective responsibility in situations involving UWSA, and the prevention of UWSA through strengthening sexuality education in schools.

### 4.1. Toward a normalisation of unwanted sexual attention in youth culture

Findings from this study indicate that the women perceived UWSA as normalised within youth culture, a view that aligns with previous Norwegian research. Sexual harassment is widespread among Norwegian adolescents, partly due to their increased engagement with social media and digital platforms (Pedersen et al., 2023). Data from the Ungdata surveys (Bakken, 2023, 2024) and The Norwegian Ombudsman for Children’s report (2018) show that young people experience sexual harassment, with girls reporting such experiences more frequently than boys. The Ungdata surveys reveal that verbal UWSA is prevalent as early as age 13-14 (Bakken, 2022, 2024). These findings are consistent with similar studies conducted in Sweden and Canada, which show that children aged 10–12 experience sexual harassment (Erentzen et al., 2023; Skoog et al., 2023; Valik et al., 2023). Although the women in this study viewed sexual harassment as unacceptable, some perceived verbal and digital UWSA as less problematic. Previous research has shown that perceptions of UWSA from peers change over time, with the threshold for recognising such behaviour as problematic increasing with age and development (Valik et al., 2023; Horn & Poteat, 2023). Students’ perceptions of the severity of sexual harassment were found to be linked to how frequently teachers intervened in such cases (Horn & Poteat, 2023).

The participants described *verbal* and *digital* UWSA as “normal” within youth culture. Even when digital UWSA was experienced as uncomfortable, they often chose non-confrontational strategies such as ignoring, deleting, or blocking the sender. This aligns with earlier findings showing that blocking is a common response, particularly among younger girls (Norwegian Media Authority, 2022; Salerno-Ferraro et al., 2022). However, the proportion of youth who block decreases with age (Norwegian Media Authority, 2024), which may reflect resignation or desensitisation. This paradox, between recognising UWSA as unacceptable and simultaneously accepting it, can be interpreted as part of an ongoing normalisation process, where the boundaries of what is considered harassment gradually shift. Previous research supports this interpretation. A research synthesis by Brown et al. (2020) on the normalisation of sexual harassment among young women found that the context of children’s upbringing and development (including parents, peers, schools, and media) contributes to a culture in which sexual harassment is tolerated, potentially increasing its prevalence among young adults (Brown et al., 2020). Societal attitudes toward sexual harassment play a central role in both its normalisation and the tendency to minimise or excuse perpetrators’ actions (Cullen-Rosenthal & Fileborn, 2023). In our study, the participants acknowledged sexual harassment as a serious societal issue and expressed support for measures promoting gender equality and respect. At the same time, they sometimes downplayed the severity of such incidents, for example by interpreting them as compliments. These discourses may undermine recognition of sexual harassment as a serious problem and weaken both prevention efforts and support for victims (Cullen-Rosenthal & Fileborn, 2023). This reflects a contradiction between society’s stated values and actual practices: while UWSA is publicly acknowledged as a problem, it is often trivialised or accepted in everyday interactions. Such inconsistency may contribute to the reinforcement of collective cultural gender stereotypes.

Boys and young men are also subjected to UWSA. A qualitative study from Canada by Erentzen et al. (2023) found that males aged 16–23 reported experiences of catcalling, seductive behaviour, unwanted touching, and requests for nude images from women, often beginning as early as ages 9–12. These young men expressed uncertainty about gender role expectations and believed they were supposed to enjoy such attention, even though they found it disturbing and uncomfortable (Erentzen et al., 2023).

Research shows that sexual harassment is frequently normalised and trivialised, especially in school contexts, where it is often framed as teasing or bullying. Boys are sometimes excused on the basis of hormonal impulses, and some girls are perceived as enjoying the attention. In some cases, boys described harassment as a form of affection or romantic interest (Magalhães et al., 2020). Some women in our study also described sexual attention as something that could boost self-esteem and be perceived as a compliment. At the same time, they acknowledged the objectification of women. This perception is linked to cultural gender stereotypes and societal expectations regarding male and female interactions. Men are expected to be sexually active, while women are objectified and valued for their sex appeal, seeking attention and prioritising men’s sexual needs (Brown et al., 2020). These complementary roles reinforce one another, and the authors argue that boys’ sexual harassment of girls is a direct consequence of gender stereotypes, much like racial discrimination stems from racial stereotypes (Brown et al., 2020). A previous study from Salmon and Brown found that comments about appearance and objectification are common forms of sexual harassment experienced by girls (Salomon & Brown, 2021). Half of the boys in that study believed such comments were socially accepted and part of romantic capital. Interventions should recognise that many boys view this behaviour as a way to express romantic interest and should focus on correcting these assumptions while promoting more respectful and prosocial forms of expression and approach (Salomon & Brown, 2021).

Women generally show lower tolerance for sexual harassment than men (Bitton & Shaul, 2013; Horn & Poteat, 2023). Research indicates that feminist identity and a critical stance toward traditional gender roles are associated with lower acceptance of sexual harassment (Shi & Zheng, 2020). The ability to recognise sexual harassment is influenced by such attitudes, and studies show that feminist-oriented education and efforts to promote gender equality play a key role in developing more nuanced and critical understandings of what constitutes sexual harassment (Shi & Zheng, 2021). These perspectives are essential in the development of preventive interventions, as attitudes toward gender and equality affect both recognition and acceptance of harassing behaviour.

### 4.2. Individual vs. collective responsibility in situations involving UWSA—the need for bystander training

The women in the study emphasised the importance of looking out for one another and having the courage to speak up and support each other in situations involving UWSA. They described how the responsibility is often perceived as individual, where the person affected is expected to act “appropriately” in the moment, while also recognising that bystanders carry a significant responsibility. This points to a tension between individual and collective responsibility in handling UWSA. A previous study showed that adolescents often refrain from speaking up because they lack trust in adults to respond appropriately (Brown et al., 2023). For individuals exposed to UWSA to feel safe enough to speak up, they must trust that those around them, both peers and adults, will support and respond in ways that protect and validate their experiences. Salomon and Brown (2021) found that many adolescents, both boys and girls, expressed a willingness to intervene in situations involving sexual harassment, motivated by moral convictions. However, the authors noted that such responses may reflect hypothetical intensions rather than actual behaviour, as young people may struggle to recognise harassment or fear that intervening could escalate the situation (Salomon & Brown, 2021). This was also reflected in our study, where some of the women expressed that speaking up or intervene can be difficult due to uncertainty and fear of escalation. Research shows that bystander training can strengthen adolescents’ ability to act in such situations. An American study found that both personal and peer-related social norms influenced how bystanders perceived sexual harassment, assessed its severity, felt responsible to intervene, and knew how to act (Nickerson et al., 2023). A youth-led intervention among students in grades 7–10 showed that helpful bystander behaviour declined over time. However, a high degree of internalised positive social norms and a low level of denial regarding sexual violence as a problem were associated with more active helping behaviour (Banyard et al., 2021).

The women in our study believed that many people hesitate to intervene in situations involving UWSA, highlighting the need for systematic bystander education. A systematic review found that bystander training can positively influence both attitudes and behaviour, especially when it includes instructions, discussion, and active learning methods (Mujal et al., 2021). Nickerson et al. (2024) found that bystander training increased adolescents’ knowledge about sexual harassment, sense of responsibility, readiness to act, and ability to support and alert adults. Students reported that the training was particularly helpful because it allowed them to practice different strategies. The authors suggested that bystander intervention training should be integrated into efforts to promote a positive school climate, with a focus on social and emotional competence (Nickerson et al., 2024). Equally important is that youth who speak up can trust that adults will take them seriously and respond in a safe and supportive manner (Brown et al., 2023).

### 4.3. How to prevent UWSA and sexual harassment through strengthening sexuality education in schools?

Findings from our study indicate a clear need for increased competence and knowledge related to sexual health, sexual rights, and especially respect and boundary-setting. Participants emphasised this need by pointing to the potentially serious consequences of UWSA. All the women in the study reported having received insufficient education on boundary-setting and called for a strengthening of sexuality education in this area. This need is also supported by previous research (The Ombudsman for Children, 2018; Goldschmidt-Gjerløw, 2022; Helbekkmo et al., 2021; Sex and Society, 2022). Knowledge about rights, respect, and boundaries is essential for developing personal autonomy while also respecting others’ limits (Norwegian Ministry of Health and Care Services, 2016). Municipalities must ensure that all children and young people have access to comprehensive sexuality education from early childhood through school (Norwegian Diractorate of Health, 2020). The women in our study believed that education on sexual health and boundary-setting should begin earlier and be repeated regularly. Research shows that sexual harassment can occur early in childhood (Erentzen et al., 2023; Skoog et al., 2023; Valik et al., 2023), supporting the argument raised by some participants, for starting sexuality education already in preschool. A systematic review also supports the need for earlier and repeated sexuality education (Goldfarb & Lieberman, 2021). Key areas for improvement in sexuality education include basic information about sex, diversity in sexuality and sexual identity, greater emphasis on the emotional and relational aspects of sex, earlier and more frequent instruction, and the provision of updated and realistic information delivered by qualified professionals (Astle et al., 2021).

Education on sexual harassment and boundaries should be strengthened. A Norwegian study found that girls in upper secondary school especially called for more competence on this sensitive topic. This need may reflect a desire for recognition and respect, rooted in experiences of disrespect and powerlessness (Goldschmidt-Gjerløw, 2022). Another Norwegian study on social-studies teachers’ practices related to teaching and preventing sexual harassment, revealed significant variation in whether the topic was addressed. Female and younger teachers were more likely to include the topic than male and older teachers. The identity of the teacher proved to be as influential as the school’s cultural environment (Goldschmidt-Gjerløw, 2022).

A cross-sectional study from the United States found that young women who felt school leadership ignored reports of sexual harassment were more likely to experience harassment from peers. This led to reduced feelings of safety and serious consequences such as emotional and behavioural withdrawal, thoughts of dropping out, increased absenteeism, difficulty concentrating, and disengagement from schoolwork (Ormerod et al., 2023). The women in our study also highlighted the psychosocial consequences of UWSA, underscoring the need for school climate initiatives to include measures against sexual harassment. School policies must be reflected in both staff and student attitudes and behaviours, and all actors must be equipped with the knowledge and skills to combat sexual harassment.

Preventing UWSA should involve students, teachers, and parents, as all are part of the social contexts in which harassment can occur (Bolduc et al., 2023). Involving youth in the development of educational programmes is essential (Sakellari et al., 2022), and a Norwegian study found that such involvement was perceived as valuable by students (Goldschmidt-Gjerløw, 2022). Training should target young people directly, as well as include adult education for parents and teachers, peer and family support, formal sanctions, and health-promoting teaching methods. Immediate action must be taken when harassment occurs, and bystanders should be encouraged to seek help from teachers and adults (Sakellari et al., 2022). This aligns with findings from our study, where young women emphasised the importance of seeking help, having the courage to intervene, and ensuring that such behaviour is sanctioned promptly.

The use of varied and student-active pedagogical methods may be necessary to prevent UWSA. Role-play exercises with well-developed scripts are one possible method (Brandslet, 2017). Implementing more student-centred approaches, such as role-play, requires teachers who are comfortable with the method (Leming, 2016). A prevention project across four European countries, in which youth were involved in developing the intervention, including role-play exercises, showed that students became more aware and willing to intervene in cases of sexual harassment. The distinction between bullying and sexualised violence was clarified, and students’ attitudes and behaviours changed. They also demonstrated increased engagement in preventing sexual harassment during and after the programme (Magalhães et al., 2020).

Promoting knowledge and awareness early may be key to preventing UWSA, but both the methods and content of education are crucial. A UK study (Ringrose & Regehr, 2023) showed that image-based sexual harassment is gendered, with girls pressured to send nude images and later blamed and shamed. The authors argued that sexuality education often reduces this issue to a moralising “don’t send pictures” message directed at girls and called for a shift in focus toward challenging boys’ attitudes and behaviours.

The women in our study believed that a lack of knowledge about boundaries, attitudes, and respect contributed to the occurrence of UWSA and sexual harassment. Regarding pedagogical methods in sexuality education and harassment prevention, it may be useful to recognise that harassment can stem from young people’s lack of social sensitivity, awkward behaviour, and unfamiliarity with boundaries and social norms. Young people therefore need clear scripts for how to express sexual interest and interpret such interest in others (Bendixen & Kennair, 2017; Brandslet, 2017). School-based interventions can help youth express romantic or sexual interest in more respectful ways (Salomon & Brown, 2021). Emphasising gender equality and feminist education is important for improving perceptions of sexual harassment (Shi & Zheng, 2021). This highlights how societal attitudes toward gender and sexual harassment play a central role in shaping and normalising UWSA. To address the cultural gender stereotypes that sustain sexual harassment, it is essential to work at both individual and structural levels. Interventions, particularly in school settings, should be made available to all. Successful prevention and education efforts must be holistic, incorporating knowledge-building, attitude change, emotional competence, the promotion of positive social norms, and the development of action competence.

## 5. Conclusion

The findings of this study highlight a clear need to strengthen education on sexual rights, boundary-setting, and practical competence in responding to UWSA among children and adolescents. Current sexuality education is often insufficient, particularly regarding topics such as respect, social norms, and sexual harassment. Schools emerge as a key arena for the prevention of UWSA. Effective prevention requires a holistic and interdisciplinary approach that combines early and continuous education, youth involvement, and student-centred pedagogical methods. Educational programmes should be relevant, inclusive, and adapted to today’s digital and social realities.

It is essential to challenge gender stereotypes and the ongoing normalisation of UWSA, while ensuring that school staff and school health services possess the necessary competence to prevent and address UWSA and sexual harassment. If the normalisation of this serious societal issue leads to underreporting among youth, it may have significant consequences for the psychosocial and sexual health of those affected. The responsibility for addressing UWSA must not be individualised; instead, a collective understanding of responsibility should be promoted. To succeed in both prevention and response, it is necessary to create safe and respectful school environments, and this work must be anchored politically and organisationally.

## 6. Contribution, strengths, and limitations of the study

This study contributes valuable knowledge about UWSA among youth by highlighting young women's perceptions and perspectives. There is limited qualitative research that centres the voices of young people, and this study helps fill a gap in the field. The findings provide insight into how UWSA is perceived, its potential consequences, and which measures are considered relevant and necessary for prevention. This offers a useful foundation for the development of school-based interventions and educational programmes that promote respect and prosocial behaviour, and that may help reduce the normalisation of harassing behaviour. Interdisciplinary collaboration, particularly between school nurses and social studies teachers, appears to be a promising approach to enhancing the quality and relevance of sexuality education.

One of the study’s strengths is its qualitative design, which allows for a deeper understanding of a complex and sensitive topic. By analysing participants’ own narratives, the study sheds light on both individual experiences and structural challenges related to sexuality education and the prevention of sexual harassment. The study is further strengthened by situating its findings within relevant national and international research, which enhances its credibility and transferability. The present manuscript was cross-checked to comply with the consolidated criteria for reporting qualitative studies using the 32-item COREQ checklist (Tong et al., 2007).

To ensure the trustworthiness of the study, we applied the criteria of credibility, confirmability, dependability and transferability, as outlined by Lincoln and Guba (1985). *Credibility* was enhanced by ensuring coherence throughout the research process, including alignment between the study’s aim, the selection of participants, the procedures for data collection and analysis. Direct quotations from the participants perspective demonstrated credibility in the interpretation of the data. *Confirmability* was enhanced through repeated discussions among the authors about the meaning units, sub-themes, and themes, ensuring that the interpretations were grounded in the data rather than in researcher preconceptions. *Dependability*, which concerns the stability and reliability of the findings and the potential for replication, was supported by detailed descriptions of the research process, including context, participant characteristics, and procedures for data collection and analysis. *Transferability* was addressed by providing thorough descriptions of the study context and participants, enabling readers to assess the applicability of the findings to other settings or groups.

However, the study also has certain limitations. The sample consists exclusively of young women, which provides a one-sided gender perspective. While this offers valuable insight into a group that is often affected by UWSA, it limits the ability to understand the phenomenon from a broader gender perspective. Future research should therefore include young men and non-binary individuals to achieve a more comprehensive understanding. Additionally, the study is based on self-reported perceptions, which may be influenced by a desire to present oneself in a socially acceptable manner. Despite these limitations, the study provides a solid foundation for further research and practice development. It points to the need for early and continuous sexuality education, interdisciplinary collaboration in schools, and the use of student-centred and innovative teaching methods. The study also emphasises the importance of understanding UWSA as a structural and cultural issue, rather than solely an individual responsibility.

## 7. Implications for future research

To strengthen the knowledge base for developing effective and targeted prevention measures against UWSA, several areas warrant further exploration. There is a need to include a broader gender perspective by investigating the perceptions and experiences of young men and non-binary individuals. Future studies should also examine the effectiveness of various teaching methods and prevention programmes in schools, with particular focus on student-centred and interdisciplinary approaches. It is recommended to explore how collaboration between different professional groups in schools, such as school nurses and social studies teachers, can enhance education on sexual health and the prevention of UWSA. Further research should also explore the views of those with previous experience of UWSA, and how digital media and social platforms influence young people's experiences and understanding of sexual harassment.

## Supplementary Material

230126 Vedlegg COREQ UWSA.pdf 230126 Vedlegg COREQ UWSA.pdf

## Data Availability

The dataset used and analysed during the current study is available from the corresponding author on reasonable request due to ethical restrictions.

## Additional information

We hereby confirm that we have read and agreed to comply with the Taylor & Francis AI Policy. In accordance with the policy, we disclose that we have used Copilot—Microsoft 365 during the preparation of the manuscript. The tool was used for editing, and the reason for its use was to enhance clarity.
